# The impact of communication training on the clinical care of hypertension in general practice: a cluster randomized controlled trial in China

**DOI:** 10.1186/s12875-024-02344-1

**Published:** 2024-03-26

**Authors:** Chuan Zou, Lili Deng, Jianzhao Luo, Hua Dai, Yu Zhang, Ru Guo, Xiaolu Luo, Rong Yang, Haiqi Song, John Spicer, Qian Zhao, Xiaoyang Liao

**Affiliations:** 1 General Practice Ward/International Medical Center Ward, General Practice Medical Center, West China Hospital, Sichuan University, NO.37 Guoxue Lane, Wuhou District, Chengdu, 611130 China; 2 The Department of General Practice, Affiliated Fifth People’s Hospital of Chengdu University of Traditional Chinese Medicine, Chengdu City, China; 3 Teaching&Research Section, General Practice Medical Center, West China Hospital,Sichuan University, Chengdu, China; 4 The Department of Gastroenterology, The First Affiliated Hospital of Chengdu Medical College, Chengdu City, China; 5 The Department of General Practice, Community Health Center of South Railway Station, Chengdu City, China; 6 Fellow of the Royal College of General Practitioners, Country Park Practice, London, UK

**Keywords:** Calgary-Cambridge guides, Primary care, Hypertension, Communication skills, Training

## Abstract

**Background:**

Hypertension is one of the most common chronic diseases with a low control rate globally. The effect of communication skills training contributing to hypertension control remains uncertain. The aim of the present study was to assess the effectiveness of an educational intervention based on the Calgary-Cambridge guide in improving hypertensive management.

**Methods:**

A cluster randomized controlled trial enrolled 27 general practitioners (GPs) and 540 uncontrolled hypertensive patients attending 6 community health centers in Chengdu, China**.** GPs allocated to the intervention group were trained by an online communication course and two face-to-face workshops based on Calgary-Cambridge guides. The primary outcome was blood pressure (BP) control rates and reductions in systolic and diastolic BP from baseline to 3 months. The secondary outcome was changes in GPs’ communication skills after one month, patients’ knowledge and satisfaction after 3 months. Bivariate analysis and the regression model assessed whether the health provider training improved outcomes.

**Results:**

After the communication training, the BP control rate was significantly higher (57.2% vs. 37.4%, *p* < 0.001) in the intervention groups. Compared to the control group, there was a significant improvement in GP’s communication skills (13.0 vs 17.5, *p* < 0.001), hypertensive patients’ knowledge (18.0 vs 20.0, *p* < 0.001), and systolic blood pressure (139.1 vs 134.7, *p* < 0.001) after 3 months of follow-up. Random effects least squares regression models showed significant interactions between the intervention group and time period in the change of GP’s communication skills (Parameter Estimated (PE): 0.612, CI:0.310,0.907, *p* = 0.006), hypertensive patient’s knowledge (PE:0.233, CI: 0.098, 0.514, *p* < 0.001), satisfaction (PE:0.495, CI: 0.116, 0.706, *p* = 0.004), SBP (PE:-0.803, CI: -1.327, -0.389, *p* < 0.001) and DBP (PE:-0.918, CI: -1.694, -0.634, *p* < 0.001), from baseline to follow-up.

**Conclusion:**

Communication training based on the Calgary-Cambridge guide for GPs has shown to be an efficient way in the short term to improve patient-provider communication skills and hypertension outcomes among patients with uncontrolled BPs.

**Trial registration:**

The trial was registered on Chinese Clinical Trials Registry on 2019–04-03. (ChiCTR1900022278).

**Supplementary Information:**

The online version contains supplementary material available at 10.1186/s12875-024-02344-1.

## Introduction

Hypertension is one of the most common chronic diseases in the world and the primary risk factor for cardiovascular diseases such as myocardial infarction or stroke [1, 2]. But only 13.8% of patients with hypertension are under control worldwide [3], while 15.3% in China are controlled [4]. Anti-hypertensive medication can effectively reduce adverse outcomes. Factors such as self-efficacy, hypertension knowledge, patient satisfaction, and medication adherence may influence patients' decisions to use anti-hypertensive drugs [5, 6]. Studies have shown that effective doctor-patient communication may indirectly improve medical outcomes by targeting these factors [7, 8]. However, randomized control trials on whether communication skills training contributes to the hypertension control effect have shown inconsistent results [9–12].

A recent meta-analysis of six experimental trials in people with hypertension concluded that interventions to improve communication were likely to improve satisfaction, but there was little effect on secondary outcomes, such as systolic and diastolic blood pressure [13]. One of the main reasons for this unclear effect was the diversity of training interventions across studies: motivational interviewing, patient-centered care, shared decision making and other communication techniques [13]. Calgary-Cambridge Guide(C-CG), developed based on evidence in medical interviews, is a comprehensive and popular communication model in many countries, which integrates various components involving active listening, gathering information, empathy, shared decision making, etc. [14]. Studies have demonstrated that the Calgary-Cambridge Guide can effectively improve health providers’ communication skills [15, 16].

General practitioners (GPs) in China, similar to family physicians or GPs in other countries, have an integral role in providing active and continuous services for hypertensive patients in primary care settings, including health education, empowering patients, adherence monitoring and medication tailoring [17, 18]. Patients with hypertension in the community form longitudinal relationships with GPs through family doctor contracting program in China [19].

Therefore, we hypothesize that communication training based on C-CG for GPs may improve patients’ hypertension knowledge and healthcare satisfaction, thus positively affecting hypertension outcomes. Besides, our previous survey revealed that Chinese GPs exhibited a significant training need for improving their communication skills, specifically in building rapport, displaying empathy, and participating in shared decision-making, all encompassing in the Calgary-Cambridge Guide [20]. This study aimed to evaluate whether C-CG-based communication training can improve the communication skills of GPs and bring a positive impact on clinical outcomes in hypertensive patients.

## Method

### Setting

This cluster randomized controlled trial adhered to CONSORT guidelines and was conducted between September 2019 and March 2021 in Wuhou District, Chengdu, Sichuan Province. Six community health centers (CHCs) out of a total of thirteen CHCs of the Wuhou District, with seven rejected due to various reasons, such as participating other research program.

### Participants

GPs were enrolled from selected CHCs if they (a) had obtained a qualified certificate for general practice, (b) provided health care services for hypertensive patients during the past year before the educational intervention and (c) agreed to be enrolled.

We recruited patients according to the ratio of 20:1 with GPs in each CHC with the following characteristics: (a) diagnosis of primary hypertension according to “Chinese Guidelines for Prevention and Treatment of Hypertension, 2018” [21];(b) aged 18 years or older; (c) prescribed at least one antihypertensive medication during the past 3 months. Patients were excluded if they (a) had secondary hypertension or had hypertensive emergency; (b) had mental disturbance, visual impairment, or mental disturbance; (c) were pregnant or lactating women.

Informed consent was obtained from all participants including the GPs and hypertensive patients.

### Randomization and blinding

The recruited 6 CHCs were allocated (1:1) to either the intervention or the control group using a random number table generated by SPSS26. Due to the nature of the educational intervention, blinding of GPs was not feasible. However, patients were blinded to the GP's group allocation, and importantly, the evaluators assessing the outcome measures were also blinded to whether the patients were in the intervention or control group.

### Intervention

The GPs in the intervention group completed six online theoretical learning sessions and two face-to-face workshops, all of which were developed by GP communication trainers based on the Calgary-Cambridge guidelines. Before the experimental study, our team consisting of 5 experienced GP trainers in communication, recorded an online communication course [22], which was developed according to the Calgary-Cambridge guide with details in Supplement I. Each session was accessed on the “Tencent Meeting App” and lasted around an hour, comprising a 30-min group viewing of instructional videos followed by a 30-min group online discussion among the GPs.

The two face-to-face workshops were scheduled for the GPs in the intervention group to practice the communication skills they had learned during the online theoretical learning sessions. The workshops were guided by a senior GP trainer, one taking place after GPs completed the first three online sessions, and the other taking place after once all six sessions had been completed. In this workshop, GPs were divided into four groups to participate in role-play exercises, interact with standardized patients—trained actors simulating real patient scenarios—and receive individualized feedback to refine their clinical and communication skills. Each workshop took 2.5 h. GPs in both intervention and comparison group were provided with the printed and electronic handbook of *“Chinese Guidelines for Prevention and Treatment of Hypertension”* [21].

### Measures and outcomes

#### Patient and GP characteristics

Patients’ sociodemographic characteristics, including gender, age, marital status, education, income, and medical insurance and GPs’ sociodemographic characteristics, including gender, age, education, work experience, and professional title, were both obtained through self-report at baseline. Patient’s clinical data were extracted from the EMR at baseline, including smoking, alcohol consumption, family history of hypertension, cardiovascular family history, comorbid conditions, diabetes, duration of diagnosis, and body mass index.

#### GP Communication skills

GP consultations with study participants by standardized patients were videotaped (Songdian 514KM/534KM; Shenzhen, China) at baseline and one month after the end of the training program. Two observers independently rated the communication skills using the validated instrument of SEGUE Framework [23]. The SEGUE Framework contains 25 items, which are classified into the five dimensions as follows: (1) Set the stage; (2) Elicit information; (3) Give information; (4) Understand the patient’s perspective; (5) End the encounter. Responses for all items range from 0 (unable to answer or no) to 1(yes) [23].

#### Hypertension knowledge

Patients’ hypertension knowledge was assessed by the Hypertension Knowledge-Level Scale (HK-LS) at baseline and 3 months follow-up. The HK-LS comprises 22 items, each a statement that respondents judge as correct or incorrect. Example items include 'Taking medication daily is necessary when blood pressure is elevated' and 'If untreated, elevated blood pressure can lead to stroke.' Scores ranging on the HK-LS is 0 to 22, with higher scores indicating higher levels of hypertension knowledge. Cronbach’s α of the overall scale was 0.82 [24].

### Patient satisfaction

Satisfaction with general practice care was assessed using the the 23-item European Task Force on Patient Evaluation of General Practice (EUROPE), validated for its reliability and applicability in China [25]. This scale allows patients to express their satisfaction across various dimensions of care, rating each item on a five-point Likert scale from ‘1 = poor’ to ‘5 = excellent’. Examples of items from this scale are 'Listening to you', 'Offering you services for preventing' and ‘Interest in your personal situation’. Data were collected at baseline and 3 months follow-up.

#### Blood Pressure

We assessed patients’ BP at baseline and 3 months follow-ups using an automatic, portable machine (Omron HEM-757), validated according to international validation protocol. Three blood pressures were collected from each participant after sitting still for 5 min. The three measurements were averaged. The blood pressure readings were conducted by a member of the research team, ensuring consistency and reliability in the measurement process. BP control was classified as uncontrolled (SBP ≥ 140 mmHg or DBP ≥ 90 mmHg, or SBP ≥ 130 mmHg or DBP ≥ 80 mmHg if diabetic or chronic kidney disease) or controlled.

#### Sample Size

To detect a clinically relevant 5mmHg reduction of systolic blood pressure between groups after 3 months follows up, with an estimated standard deviation of 12.4mmHg based on previous communication researches [9, 26, 27] and 80% power at a 5% significance level, a minimum of 97 hypertensive patients per group was needed. With an inflation factor of 1.95 for the cluster design, assuming a cluster size of 20 patients and an intra-class correlation coefficient of 0.05 (for GP level) [28, 29], 190 patients per group were needed. To allow for 10% attrition, the arm was to include 211 patients in each group.

### Statistical analysis

Analyses were conducted using SPSS version 26 (Chicago, IL, USA). All outcomes were analyzed according to the intention-to-treat principle (ITT) [30]. The variable’s primary features were described as mean, standard deviation (± SD), median, interquartile range (IQR) or proportions (%). First, we used bivariate analyses (T-Test, chi-square or Mann–Whitney U) to test significant differences between clinical characteristics and socio-demographics in intervention and control groups in Table 2.

We conducted the random-effects least squares regression model to account for clustering patients within GP. This model included the main effects of study arm assignment (control vs. intervention), period (baseline vs. follow-up), their interaction and adjusted the patient demographics characteristics. SPSS 22 (Chicago, IL, USA) and R version 3.0.2 produced accurate estimates. A *P* value of 0.05 was considered to be significant.

## Results

### GPs and patients’ characteristics

**Fig. 1:**
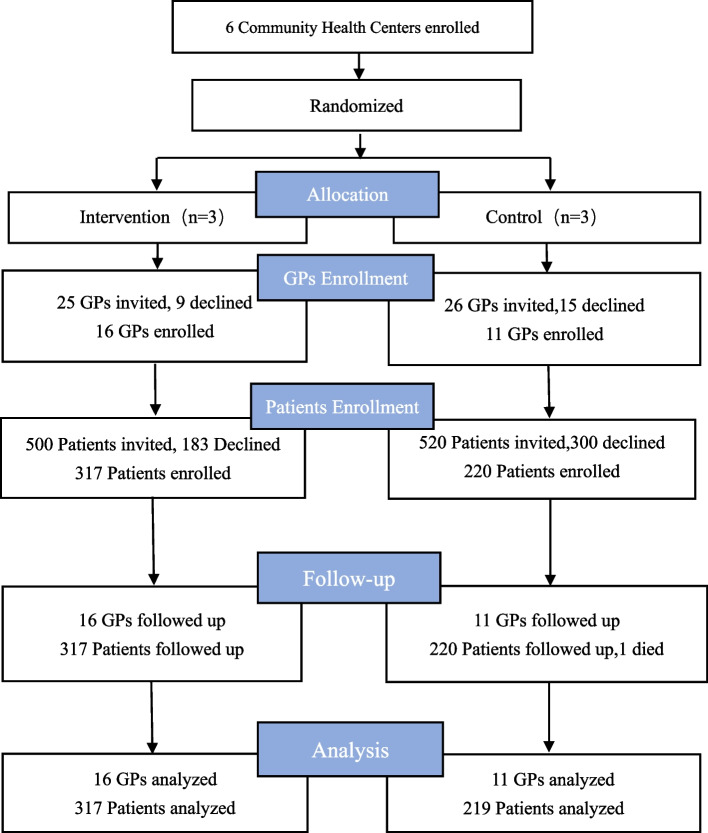
CONSORT flow diagram of the study

Figure 1 describes the flow of GPs and patients’ completeness of data. A total of 27 GPs were enrolled in the training program. Most GPs were female (62.96%), with a median age of 40 and more than ten years of work experience at selected six community health centers (Table 1). The hypertensive patients enrolled onto the study had a mean age of 64.81 ± 10.74 years and were mostly male (52.78%), married (87.78%), had less than junior high school education (61.11%), and had a median income of 3000 yuan monthly. Clinically, some patients also had a diagnose of diabetes (35.93%) and other comorbid conditions (20.19%), had a median time since hypertension diagnosis of 7 years, family history of hypertension (48.3%) and cardiovascular events (6.85%). (Table 1). At baseline, there was no difference in the total score of GP communication skills and the hypertension knowledge, medication adherence, systolic blood pressure of patients in both study groups. However, there were significant differences in GPs’ age ( *p* < 0.001), and patients’ income (*p* = 0.001), cardiovascular family history (*p* = 0.035), satisfaction with health care (*p* < 0.001) and diastolic blood pressure (*p* < 0.001) of hypertensive patients (Tables 1 and 2).

**Table 1:** Baseline characteristics of general practitioners and patients

Characteristics	Total	Intervention	Control	*P*-value
GPs(n)	*n* = 27	*n* = 16	*n* = 11	
Female (%)	17(62.96)	11(68.75)	6(54.55)	0.687
Age, M(IQR), years	40.00(36.00, 43.00)	36.00(34.25, 40.75)	44.00(43.00, 49.00)	< 0.001
Bachelor’s degree or above (%)	19(70.37)	12(75.00)	7(63.64)	0.675
Working experience, M(IQR), years	10.00(8.00, 16.00)	10.00(5.25, 15.00)	11.00(10.00, 18.00)	0.112
Attending physician, n (%)	18(66.67)	12(75.00)	6(54.55)	0.535
Patients	*n* = 540	*n* = 320	*n* = 220	
Male, n(%)	285(52.78)	164(51.25)	121(55.00)	0.391
Age, Mean(SD), years	64.81(10.74)	64.95(10.79)	64.59(10.69)	0.701
Married, n(%)	474(87.78)	276(86.25)	198(90.00)	0.414
≤ High school, n(%)	330(61.11)	187(58.44)	143(65.00)	0.217
The income per month, M(IQR)	3000.00 (2000.00,5000.00)	3500.00(2000.00,5000.00)	3000.00(2000.00,4015.00)	0.001
Smoking, n(%)	96(17.78)	53(16.56)	43(19.55)	0.373
Alcohol consumption, n(%)	132(24.44)	80(25.00)	52(23.64)	0.717
Family history of hypertension, n(%)	261(48.33)	145(45.31)	116(52.73)	0.090
Cardiovascular family history, n(%)	37(6.85)	28(8.75)	9(4.09)	0.035
Comorbidity, n(%)	109(20.19)	60(18.75)	49(22.27)	0.316
Diabetes, n(%)	194(35.93)	112(35.00)	82(37.27)	0.589
Duration of diagnosis, M(IQR)	7.00(4.00,12.00)	7.00(4.00,12.00)	6.00(3.00,11.00)	0.136
BMI, Mean(SD), Kg/m^2^	25.37(3.13)	25.16(3.18)	25.68(3.03)	0.058

*Abbreviations*: *SD* Standard deviation, *M(IQR)* Median (inter quartile range), *BMI* Body mass index

**Table 2:** Unadjusted baseline vs. follow-up scores for each outcome

Variables	Baseline			Follow up			Variation from baseline to follow up		
	Intervention	Control	*P*-value	Intervention	Control	*P*-value	Intervention	Control	*P*-value
GPs outcomes(*n* = 27)									
Communication skills Total score, M(IQR)	14.00(12.00, 15.00)	13.00(12.00, 15.00)	0.818	17.50(14.25, 19.75)	13.00(11.00, 14.00)	< 0.001	2.50(2.00, 5.75)	0.00(-2.00, 2.00)	< 0.001
Interviewing length, M(IQR), seconds	605.00(487.50, 976.50)	623.00(533.00, 825.00)	0.855	605.50(375.00, 959.50)	657.00(534.00, 761.00)	0.707	-7.50(-172.00, 103.25)	78.00(-211.00, 129.00)	0.451
Patients’ outcomes(*n* = 540)									
Hypertension knowledge, M(IQR)	18.00(17.00,19.00)	18.00(15.00,20.00)	0.082	20.00(17.00, 20.00)	18.00(18.00, 21.00)	< 0.001	2.00(0.00, 4.00)	0.00(-1.00, 2.00)	< 0.001
Satisfaction, Mean(SD)	92.11(7.06)	95.14(9.85)	< 0.001	94.98(6.89)	94.57(8.17)	0.539	2.87(7.47)	-0.57(8.50)	< 0.001
SBP, Mean(SD), mmHg	146.98(8.53)	147.40(10.83)	0.614	134.75(12.40)	139.13(11.58)	< 0.001	-12.23(13.21)	-8.27(13.97)	0.001
DBP, Mean(SD), mmHg	82.10(8.50)	85.91(8.53)	< 0.001	78.33(8.70)	80.74(8.56)	0.002	-3.76(9.48)	-5.17(9.87)	0.096
BP control rate, n(%)	––	––	––	183.00(57.2)	69.00(31.4)	< 0.001	––	––	––

Communication skills, (SEGUEset elicit give understand end framework ); Knowledge, Hypertension Knowledge-Level Scale (HK-LS); Satisfaction, European Task Force on Patient Evaluation of General Practice (EUROPE)

*Abbreviations*: *SEGUE* Set elicit give understand end framework, *M(IQR)* Median (inter quartile range), *SD* Standard deviation, *SBP* Systolic Blood Pressure, *DBP* Diastolic Blood Pressure, *BP* Blood Pressure

### Educational intervention outcomes

We found that GP’s communication skills significantly differed in the change of total communication scores from baseline to follow-up in the intervention compared to the control group (Median: 2.50 vs 0.0, *p* < 0.001). However, the duration of clinical encounters demonstrated no significant differences (Median: -7.50 vs 78.00, *p* = 0.451).

After the intervention, BP control rate was significantly increased (57.2% vs 37.4%, *p* < 0.001). A significant difference was found between hypertensive patients of intervention versus control groups at follow-up and in change from baseline to follow-up in all the scores, including hypertension knowledge, medication adherence, and SBP (Table 2).

These results were in agreement with the random effects least squares regressions models which showed significant interactions between intervention group and time period. Thus, we found significant evidence in the change in GP’s communication skills (Parameter estimated(PE):0.612, CI:0.310,0.907, *p* = 0.006), hypertensive patient’s knowledge (PE:0.233, CI: 0.098, 0.514, *p* < 0.001), satisfaction (PE:0.495, CI: 0.116, 0.706, *p* = 0.004), DBP (PE:-0.918, CI: -1.694, -0.634, *p* < 0.001), from baseline to follow-up, in the intervention compared to the control group (Table 3).

**Table 3:** Interaction of intervention with the period for each outcome

Outcome	Parameter estimated (PE)	95% *CI*	*P*-value
GPs^a^ (*n* = 27)			
Communication skills	0.612 (0.178)	0.310, 0.907	0.006
Patients^b^ (*n* = 540)			
Hypertension knowledge	0.233 (0.061)	0.098, 0.514	< 0.001
Satisfaction	0.495 (0.127)	0.116, 0.706	0.004
SBP (mmHg)	-0.803 (0.200)	-1.327, -0.389	< 0.001
DBP (mmHg)	-0.918 (0.243)	-1.694, -0.634	< 0.001

^a^Adjusted for gender, age, years providing care, and education

^b^Adjusted for gender, age, marital status, education, income per month, smoking, drinking, family history of hypertension, cardiovascular family history, comorbid conditions, diabetes, duration of diagnosis, BMI

Communication skills, set elicit give understand end framework (SEGUE); Knowledge, Hypertension Knowledge-Level Scale (HK-LS); Satisfaction, European Task Force on Patient Evaluation of General Practice (EUROPE)

## Discussion

We found that brief communication training based on Calgary-Cambridge guides for GPs could directly impact hypertensive patients’ knowledge and satisfaction over a 3 month period. Moreover, the intervention may have an indirect effect on hypertensive outcomes in the short term (e.g., SBP, DBP and BP control rate). Our findings also included improving providers’ communication skills while not extending consultation time after the education program.

Patients with hypertension typically communicate with GPs several times a year following diagnosis. Thus, the quality of these encounters can be a significant determinant of the quality of their clinical outcomes. Our findings verify the hypothesis proposed by Street [7] that patient-provider communication can directly impact patients’ BP. However, it often operates indirectly through proximal and intermediate outcomes, such as patient understanding, satisfaction, and treatment adherence [7, 31]. These results are in line with recent RCTs, which demonstrate that educational communication programs have had an impact on patient’s clinical outcomes in primary care settings after six months of follow-up, including reduction of systolic blood pressure and improvement of medication adherence [9, 26].

However, several similar studies failed to show positive results [11, 12, 32]. It is noteworthy that these studies either had longer follow-up times, ranging from 12 to 20 months, or shorter training lengths with 4–6 h compared to studies with positive findings [9]. It’s likely that the effects of “low intensity” intervention could not be sustained over time [33]. Our findings also contrasted with recent systematic reviews [13], including 6 RCTs on hypertension that showed communication skills training interventions for healthcare professionals did not improve BP control or other relevant patient outcomes. Due to the diversity of training intervention, training theory, training methods, trainers, training assessment, training length and follow-up time, the pooled results should be treated cautiously [13]. In addition, communication training methods of included studies contained motivational interviewing, patient-centered care and shared decision-making. However, our study was based on the Calgary-Cambridge guide, which proved to be evidenced, comprehensive and integrated with interviewing content and process [14]. It contains multiple communication elements, such as patient-centered care, empathy, and shared decision making, which might be the reason why the Calgary-Cambridge communication model could potentially alter medical outcomes in our study within three months.

Consistent with the findings of similar intervention studies [9, 12], our study found that GPs who received communication training were able to significantly enhance patient satisfaction and knowledge regarding hypertension management. This aligns with observations from a cross-sectional study [34] that highlighted hypertensive patients' appreciation for physicians' communication behaviors such as 'active listening,' 'speaking in a way the patient can understand,' and 'paying attention to the patient.' These behaviors foster a deeper patient involvement and shared decision-making, which are crucial for effective self-care. Two hypertensive studies [35, 36] found a positive correlation between physician–patient relationship satisfaction and medication adherence. Although direct measures of medication adherence were not included in our study's results, the emphasis on communication skills in our intervention likely serves as a mediator by improving patient engagement and potentially influencing better self-care practices. This suggests that well-informed patients, who feel supported by their healthcare providers, may be more motivated to adhere to treatment regimens, thus indirectly contributing to improved blood pressure control.

### Strengths and limitations

This is the first randomized controlled trial to estimate the potential impact of Calgary-Cambridge guides’ communication training for GPs on health outcomes in the hypertensive population. Besides the novel but generic training contents, the interventions incorporated several successful features of previous educational interventions, including multiple training methods and moderate training intensity in a relatively long period, which could potentially help maintain the training effect for GPs [13]. The intervention may still be effective during global pandemics, if training were to be delivered fully online. Thus, the training could be easier to encourage widespread implementation and potentially become a scalable approach. Further, our work documents the relationships between main covariates such as patients’ satisfaction, hypertension knowledge, and medication adherence that could help to explain better a modifiable mechanism between effective providers–patient communication and BP control for hypertensive patients [7, 31].

Some limitations are worth noting in this study. First, there was evidence of an imbalance between the intervention and control groups due to cluster randomization at baseline, both in GPs and patients. This may reflect post-randomization recruitment bias and reduce the power of detecting intervention effects. To minimize these effects, we used random effects, least squares, and regression models. Also, we included a wide range of factors that we considered may be associated with hypertensive outcomes and adjusted for potential confounders. Furthermore, this trial only had a small number of practices (6 clusters) from one region. GPs who took part were self-selected and thus likely to be more interested in communication. The external validity of our results may be limited, and the results should be interpreted with caution without further validation of these findings. On the other hand, it could be considered that GPs’ commitment to the subject matter is always necessary for effective learning. The third limitation is that we failed to collect and compare the hypertensive medications taken by both groups, especially after the intervention, which could be a confounding factor influencing blood pressure.

## Conclusion

Communication training for GPs based on the Calgary-Cambridge guide could not only enhance patient-provider communication skills, but also altered satisfaction, hypertension knowledge, and blood pressure control in the short term. Our training program provided a feasible and evidence-based method in Chinese primary care settings in hypertension management and it should be encouraged as a method of continuing professional development. Future long-term follow-up studies are required to determine whether the effects are sustainable and lead to reduced cardiovascular outcomes. In the meantime, this generic communication training could be implemented in general practice to other similar chronic diseases, such as diabetes and asthma, which need to be examined in future studies.

### Supplementary Information

**Supplementary Material 1.**

## Data Availability

The datasets generated and/or analyzed during the current study are not publicly available due to confidentiality and ethical reason but are available from the corresponding author upon reasonable request.

## Abbreviations

GPs: General practitioners
C-CG: Calgary-Cambridge Guide
CHC: Community health centers
HK-LS: Hypertension Knowledge-Level Scale
EUROPEP: European Task Force on Patient Evaluation of General Practice
SD: Standard deviation
IQR: Inter quartile range
SBP: Systolic Blood Pressure
DBP: Diastolic Blood Pressure
BP: Blood Pressure
SEGUE: Communication skills of set elicit give understand end framework
