# Supplementary material for: The impact of communication training on the clinical care of hypertension in general practice: a cluster randomized controlled trial in China
Source: BMC Prim Care. 2024 Mar 26;25:98. doi: 10.1186/s12875-024-02344-1 (PMC10964506; doi:10.1186/s12875-024-02344-1)
Supplement: Supplementary file 1 — Supplementary Material 1. [file 12875_2024_2344_MOESM1_ESM.docx]

**SUPPLEMENT**

| SUPPLEMENT I :The curriculum of offered online communication course | | | |
| --- | --- | --- | --- |
| Chapter | Topic | Demonstration | Time |
| The Value of Doctor-Patient Communication | 1. Value of doctor-patient communication: Implications from clinical evidence | Theoretical teaching | 6 Minutes |
| The structure of Calgary-Cambridge guides | 1. Introduction and the Structure of Calgary-Cambridge guides | Theoretical teaching | 5 Minutes |
| Initiating the Session | 1. Preparation | Theoretical teaching  Demonstration of interviewing simulated case  Lecturer feedback | 23 Minutes |
|  | 1. Establishing initial report |  |  |
|  | 1. Identifying the reasons for the consultation |  |  |
| Gathering Information | 1. Biomedical Perspective *vs* Patient’s Perspective | Theoretical teaching  Demonstration of interviewing simulated case  Lecturer feedback | 43 Minutes |
|  | 1. Reason for attendance, Ideas, Concerns, Expectations |  |  |
|  | 1. Techniques of Gathering information: Listening, Non-verbal skills, Open question, Summary |  |  |
| Explanation | 1. Ask-Tell-Ask-Rehearse model | Theoretical teaching | 12 Minutes |
|  |  | Demonstration of interviewing simulated case  Lecturer feedback |  |
| Planning | 1. Shared decision making | Theoretical teaching  Case presentation and feedback | 11 Minutes |
| Building the Relationship | 1. Meaning and value of building good relationships. | Theoretical teaching  Demonstration of interviewing simulated case  Lecturer feedback | 37 Minutes |
|  | 1. How to demonstrate empathy in consultation？ |  |  |
|  | 1. Developing acceptance and rapport |  |  |
| Closing the session | 1. Effective strategies for closing the session. | Theoretical teaching | 18 Minutes |
|  | 1. Forward Planning and Establishing safety-netting | Demonstration of interviewing simulated case  Lecturer feedback |  |
| Summary | 1. Case Demonstration | Demonstration of interviewing simulated case | 12 Minutes |
|  | 1. Application of Calgary-Cambridge Guides in Interview | Theoretical teaching |  |
